# Fishing for causes and cures of motor neuron disorders

**DOI:** 10.1242/dmm.015719

**Published:** 2014-07

**Authors:** Shunmoogum A. Patten, Gary A. B. Armstrong, Alexandra Lissouba, Edor Kabashi, J. Alex Parker, Pierre Drapeau

**Affiliations:** 1 Department of Neuroscience, FRQS Groupe de Recherche sur le Système Nerveux Central and CRCHUM, University of Montréal, Montréal, QC H3A 2B4, Canada.; 2 Institut du Cerveau et de la Moelle Épinière, Centre de Recherche, CHU Pitié-Salpétrière, 75013 Paris, France.

**Keywords:** ALS, HSP, SMA, Zebrafish, Drug discovery, Motor neuron disorders

## Abstract

Motor neuron disorders (MNDs) are a clinically heterogeneous group of neurological diseases characterized by progressive degeneration of motor neurons, and share some common pathological pathways. Despite remarkable advances in our understanding of these diseases, no curative treatment for MNDs exists. To better understand the pathogenesis of MNDs and to help develop new treatments, the establishment of animal models that can be studied efficiently and thoroughly is paramount. The zebrafish (*Danio rerio*) is increasingly becoming a valuable model for studying human diseases and in screening for potential therapeutics. In this Review, we highlight recent progress in using zebrafish to study the pathology of the most common MNDs: spinal muscular atrophy (SMA), amyotrophic lateral sclerosis (ALS) and hereditary spastic paraplegia (HSP). These studies indicate the power of zebrafish as a model to study the consequences of disease-related genes, because zebrafish homologues of human genes have conserved functions with respect to the aetiology of MNDs. Zebrafish also complement other animal models for the study of pathological mechanisms of MNDs and are particularly advantageous for the screening of compounds with therapeutic potential. We present an overview of their potential usefulness in MND drug discovery, which is just beginning and holds much promise for future therapeutic development.

## Introduction

Motor neuron disorders (MNDs) are characterized by progressive loss of motor neurons of the spinal cord (‘lower motor neurons’) or motor neurons of the brain (‘upper motor neurons’), or both, leading to atrophy and/or spasticity of the associated musculature. Spinal muscular atrophy (SMA), amyotrophic lateral sclerosis (ALS) and hereditary spastic paraplegia (HSP) are the most common MNDs, and we summarise their aetiologies and genetics. We then present a critical overview of the methods available to model genetic mutations in zebrafish as well as their limitations. In the main body of the article, we highlight zebrafish models for each of these MNDs and discuss recent advances in developing drug screens, with a special focus on ALS.

SMA is a neurodegenerative disease presented clinically by progressive degeneration of lower motor neurons in the anterior horn of the spinal cord, resulting in hypotonia, muscle atrophy, paralysis and, in severe cases, death (Khaniani et al., 2013). It is a genetically heterogeneous disorder, with most cases displaying a recessive inheritance; however, autosomal dominant and X-linked inheritance have been reported (Greenberg et al., 1988; Jiang et al., 2013). Over 20 studies with zebrafish models of SMA have been published to date and, out of the MNDs, stable genetic models are most advanced for SMA because many of the causative mutations cause loss of function, which, as described below, can be readily studied in zebrafish.

ALS is a late-onset neurodegenerative disorder that affects both upper and lower motor neurons and is the most common MND. Although the majority of ALS cases are sporadic, up to 10% are inherited, with mutations in over two-dozen genes accounting for the majority of familial causes (Robberecht and Philips, 2013). The recent explosion in ALS genetics has attracted many researchers to the field, and zebrafish models have been the first animal models developed for several of these genes, for which some have been advanced to therapeutic screening and drug discovery.

HSP is the collective term for a group of clinically and genetically heterogeneous neurodegenerative disorders characterized by progressive spasticity and weakness in the lower limbs due to loss of upper motor neurons (Harding, 1983). The clinical heterogeneity of HSP is related to a notable genetic heterogeneity; however, insight into the genetic basis of these disorders is expanding rapidly (Dion et al., 2009; Finsterer et al., 2012) and supports a close genetic link between HSP and other neurodegenerative diseases (Novarino et al., 2014). Zebrafish models for the disease have been presented in numerous publications and have been used to provide insights into the pathological mechanisms underlying the disease.

## Common pathological pathways between the different MNDs

**Fig. 1.**
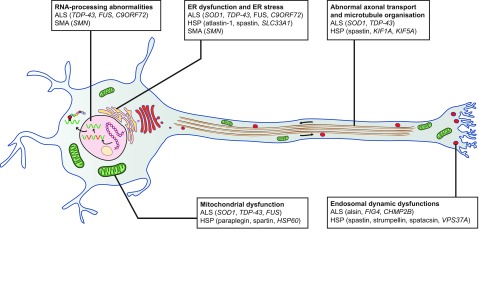
**Common pathological pathways in ALS, SMA and HSP.** An illustration of a motor neuron, highlighting common possible mechanisms in the literature linking the MNDs. A non-exhaustive list of genes involved is given for each disease.

Although MNDs have quite different aetiologies, they are all typified by progressive paralysis resulting in severe disability. The pathogenesis of MNDs remains to be fully understood and there is a crucial need for the development of novel therapeutic strategies for their treatment. A thorough understanding of disease pathophysiology is necessary to direct rationale design of therapeutic strategies. As mentioned above, MNDs are characterized by the degeneration of upper and/or lower motor neurons and this is due to a dysfunction of several different cellular processes. Despite the apparent heterogeneity in these MNDs (considered below in greater detail for SMA, ALS and HSP in turn, where we describe the major causative genes), several pathogenic pathways are common among at least two of these three diseases (summarized in Fig. 1). For instance, in both SMA and ALS, defects in RNA metabolism are implicated, which mainly lead to splicing anomalies in the nucleus and mRNA transport abnormalities in the cytoplasm. These defects have been implicated by mutations studied in zebrafish models of SMA (Akten et al., 2011; Lotti et al., 2012; Sleeman, 2013), in other models of SMA (Sleeman, 2013) and in various models of mutations in ALS (Ling et al., 2013; Sleeman, 2013). Additionally, ALS-related RNA foci sequester RNA-binding proteins and lead to possible RNA processing abnormalities (Gendron et al., 2014). Interestingly, some ALS genes have been shown to interact with genes in SMA and lead to an altered distribution of splicing factors (Achsel et al., 2013). However, no involvement of RNA metabolism has been reported in HSP.

RNA-processing genes implicated in ALS also lead to abnormal axonal transport and microtubule organisation (Godena et al., 2011; Ilieva et al., 2009), and an actin-binding protein was shown to rescue the phenotype in a zebrafish model of SMA (Oprea et al., 2008; Hao et al., 2012; Lyon et al., 2014). This has also been implicated in HSP, where spastin has been shown to be involved in microtubule severing, whereas other less common HSP genes are involved in axonal transport (Blackstone, 2012). Furthermore, many HSP genes are involved in endosomal dynamics (Blackstone, 2012), and dysfunction of endosomal dynamics has been reported for several less-common ALS-associated genes (Chen et al., 2013).

Endoplasmic reticulum (ER) stress as well as mitochondrial dysfunction have been reported as important pathological mechanisms in MNDs. For example, the inclusions found in ALS are thought to be associated with misfolding of mutant proteins and an inability of the unfolded protein response to dispose of them (Matus et al., 2013). Moreover, ER stress has been implicated in zebrafish models of ALS (Vaccaro et al., 2013), and ALS- and SMA-related proteins are important for stress granule formation (Matus et al., 2013). Several HSP genes are also involved in ER morphology and function (Blackstone, 2012). On the other hand, many HSP genes are also involved in mitochondrial dysfunction (Blackstone, 2012), as are ALS genes (Cozzolino and Carrì, 2012).

## The zebrafish toolbox: how useful is it for studying MNDs?

In recent years, the zebrafish (*Danio rerio*) has received favourable attention from clinicians owing to its advantages as a model system for the study of disease, because the zebrafish models often closely resemble the relevant human disease (Lieschke and Currie, 2007). Importantly, more than 80% of zebrafish genes share a high level of synteny across vertebrate species, in addition to 50–80% amino acid identity with most human homologues, including homologues for over 80% of disease-causing genes (Howe et al., 2013). The majority of these genes can be manipulated by loss- or gain-of-function approaches in simple transient assays of gene function and by the creation of stable disease models using genome-editing techniques. This unique ‘toolbox’ makes the zebrafish model system valuable for the study of MNDs, as discussed below.

The genetics underpinning MNDs have undergone a revolution in the last decade. Much of this can be attributed to recent advances in sequencing technologies that have greatly increased the speed and reduced the costs associated with genotyping and identifying variants in individuals afflicted with disease. Nonetheless, validating the pathogenicity of identified variants remains a challenge in human genomics for the identification of causative mutations. Although considerable efforts have been made to generate mammalian models of some of the described disease-causing mutations, zebrafish have offered an inexpensive and rapid alternative to the costly and labour-intensive requirements that accompany the creation of murine models of neurodegenerative disease. This is particularly true for some of the recently identified disease-causing genes associated with MNDs (Table 1). We note that, although no animal model, be it zebrafish or mouse, can reproduce and thus accurately model all of the subtleties of a human disease and thus are not necessarily disease models, these genetic models do reflect important molecular, cellular and even physiological features that are pertinent to disease pathobiology and pharmacological screening.

**Table 1.**
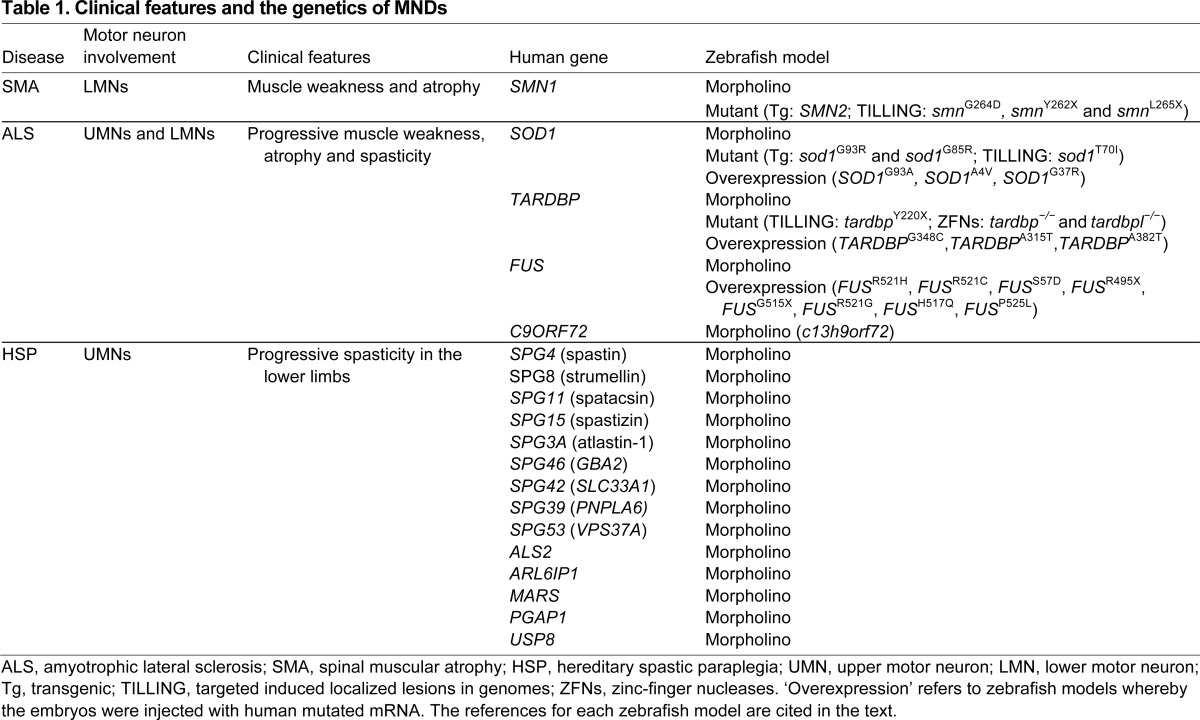
Clinical features and the genetics of MNDs

The easiest approach and therefore usually the first taken to study loss-of-function mutations in zebrafish is the injection of antisense morpholino oligonucleotides. An important caveat of the approach is that knockdown needs to be controlled for off-target toxicity by testing for the lack of effect with mismatched morpholinos and, most importantly for disease models, by demonstrating at least partial (hypomorphic) rescue by the homologous human mRNA. Furthermore, the technique is not appropriate for studying late-onset-disease-related phenotypes such as in many MNDs. Nonetheless, transient knockdowns can be useful for drug testing pertinent phenotypes at earlier embryonic and larval stages, and their advantage for high-throughput screening lies in this area (Phillips and Westerfield, 2014). Recently, powerful genome-editing techniques have been developed to generate stable loss-of-function models for neurodegenerative diseases (Schmid and Haass, 2013). These techniques have their own disadvantages because some are complex and expensive [e.g. TILLING (targeting induced local lesions in genomes) and ZFNs (zinc-finger nucleases)], or can carry their own off-target toxicity [e.g. CRISPR (clustered regularly interspaced short palindromic repeats)/Cas], such as transcriptional and post-transcriptional alterations (Varshney and Burgess, 2014). Nonetheless, genome-editing techniques have been used successfully to generate useful zebrafish models of MND, particularly in studies of SMA.

Gain of function is most easily modelled by mRNA injections, either to test for phenotypes induced by mutant alleles (dominant-negative or toxic gain of function, with the former but not the latter rescued by wild-type mRNA), or rescue of gene-knockdown or -knockout phenotypes (to test for loss of function). As with morpholinos, the transient expression of injected mRNA is not useful for late-onset phenotypes but can be quite useful for drug screening in embryos and larvae. Stable transgenesis for the study of gain-of-function mutations is a popular approach in zebrafish using the efficient *Tol2* transposon system (Suster et al., 2009). However, it requires out-crossing over several generations to isolate lines with ideally single insertions and neither too little nor too much transgene expression. Interestingly, a number of promoters are available for neural-specific expression and their inclusion in *Tol2* constructs can be used to create transgenic lines with cell-specific expression patterns (Kabashi et al., 2011b). Several MNDs have been modelled in zebrafish by the introduction of human transgenes, but stable transgenic models also have their limitations, the three major ones being ectopic expression, toxic overexpression and variability due to the genetic background (see Kabashi et al., 2011b). New and possibly simpler strategies have come from recent genome-editing approaches and many MND models using these new approaches are being developed, although no gain-of-function models have been published to date. These are particularly difficult to make because stable transgenic or genomically edited genotypes, even when heterozygous, could prove lethal, so conditional mutations might have to be engineered.

Despite some of these limitations, particularly with regard to recapitulating dominant mutations and missense mutations, the techniques currently available have been used with great success to replicate several aspects of some MNDs, as discussed in detail below. Together with site-specific transgenesis using PhiC31 (Roberts et al., 2014), Cre-*loxP* (Lin et al., 2013), the *Gal4/UAS* system (Halpern et al., 2008) and a range of inducible systems that are amenable for use in the model, these genomic engineering tools will accelerate the development of MND models in the zebrafish.

## Zebrafish as a tool for drug screening

Animal models of a disease can be used as tools for therapeutic discovery of compounds that suppress the disease phenotype, regardless of the specific molecular targets. In the last few years, it has become apparent that zebrafish is a powerful model organism amenable to high-throughput drug screening *in vivo* at embryonic and larval stages (North et al., 2007; White et al., 2011; Zon and Peterson, 2005). Thus, this model organism is becoming valuable in the preclinical pipeline to bridge the gap between *in vitro* assays and more costly screens in mammals (Giacomotto and Ségalat, 2010; Lieschke and Currie, 2007). Several studies have explored the potential of zebrafish as a model system for drug screening campaigns for cardiovascular diseases (Chan and Mably, 2011; Chico et al., 2008; Asnani and Peterson, 2014), new cancer therapies (Liu and Leach, 2011b; Stoletov and Klemke, 2008; Tat et al., 2013) and neural disorders (Baraban et al., 2013; Flinn et al., 2008; Gerlai, 2010; Vaccaro et al., 2013; Vaccaro et al., 2012a). Below we review each of the available models for the three most common MNDs as well as recent progress in developing drug discovery approaches.

## Spinal muscular atrophy (SMA)

### Background

SMA is a currently incurable neurodegenerative disease characterised by the loss of lower motor neurons, resulting in system-wide muscle atrophy (Hamilton and Gillingwater, 2013). SMA is primarily caused by deletion of the survival motor neuron 1 (*SMN1*) gene (Lefebvre et al., 1995). Clinically, SMA due to SMN1 mutation is classified as type I (severe), II (intermediate), III (mild) or IV (adult-onset) based on disease onset and severity. The *SMN2* gene is an almost identical copy of *SMN1*, and both genes encode the protein SMN (Lefebvre et al., 1995). The severity of the disease also depends on the amount of functional SMN protein produced by *SMN2* and is thus dependent on *SMN2* copy number (Hamilton and Gillingwater, 2013), with none or a single copy in type I SMA and increasing to four or more copies in type IV SMA. Zebrafish studies have primarily focused on *SMN1* because the gene can be readily studied using loss-of-function approaches.

SMN is a ubiquitously expressed nuclear and cytoplasmic protein. It is part of a multiprotein complex localised to the nuclear Gem (for Gemini of coiled bodies) bodies and is necessary for the assembly of small nuclear ribonucleoproteins (snRNPs), a central element of the spliceosome. These Gem bodies are lost in SMA, thus indicating altered splicing as a possible cause for the disease (Sleeman, 2013). Additionally, low levels of SMN seem to disrupt the cellular localisation of some mRNAs and this could be due to a possible role of SMN in the formation and/or function of messenger ribonucleoproteins (mRNP) responsible for transporting mRNAs to distal compartments of the cell (Sleeman, 2013).

### Zebrafish models of SMA

In zebrafish larvae, knockdown of *smn* to levels similar to those observed in humans with SMA results in truncated and under-branched ventral root axons and impairments in pathfinding to target musculature (McWhorter et al., 2003). The transient *smn* morphant model can be rescued by wild-type human *SMN* but not disease-causing forms (Carrel et al., 2006). Interestingly, weaker mutations rescued the phenotype at a higher dose, highlighting the sensitivity of the zebrafish system (Workman et al., 2009).

In a subsequent study, three putative *smn* mutations were generated by TILLING (Boon et al., 2009). Two mutations encoded premature stop codons (Y262X, L265X) and resulted in a deletion of the C-terminus, whereas the third encoded a point mutation (G264D) that corresponded to a human mutation associated with a severe form of SMA. Homozygous larvae expressing the Smn protein with the premature stop codon had reduced Smn protein levels by 7 days post-fertilisation (dpf) and died within 9–16 dpf. Homozygous carriers of the point mutation also displayed reduced Smn protein levels but died over a longer timeframe (13–21 dpf). In addition to the early mortality associated with loss of Smn protein expression, neuromuscular junctions (NMJs) from homozygous mutants displayed loss of colocalisation of pre- and post-synaptic markers. Transgenic expression of human SMN in motor neurons slightly increased survival in one of the homozygous mutants expressing the truncated Smn protein and recovered colocalisation of NMJ markers, indicating a cell-autonomous role for SMN in motor neurons (Boon et al., 2009). SMN mutants transgenically expressing human *SMN2* (for which there is no zebrafish homologue) also confirmed that the human *SMN2* gene is spliced in zebrafish and that increasing expression from this transgene can increase survival (Hao et al., 2011). Maternal Smn was removed from *smn* mutants so that early defects in motor neurons could be examined. Maternal:zygotic *smn* mutants were generated and revealed defects in axonal and dendritic branches as well as movement defects, indicating that SMN is crucial for motor neuron development (Hao et al., 2013). By conditionally driving the human *SMN2* gene in these animals by using the heat-shock promoter, it was shown that SMN is needed soon after motor neurons are born to rescue their development (Hao et al., 2013). These experiments show the utility of zebrafish models to test both the temporal and spatial requirement for a particular disease gene. Importantly, these transgenic and mutant approaches recapitulated the morphant phenotypes and thus support the knockdown approach for this gene. Moreover, the zebrafish line generated by Hao et al. represents a useful tool for screening for molecules that affect *SMN2* splicing (Hao et al., 2011).

More recent studies have investigated underlying pathogenic mechanisms in these SMA models. Overexpression of progranulin, whose loss of function (by nonsense-mediated decay) causes frontotemporal lobar degeneration, rescued the shortened motor neuron phenotype upon *smn* knockdown in zebrafish (Chitramuthu et al., 2010). Plastin 3, an actin-binding and bundling protein identified as a protective modifier of SMA (Oprea et al., 2008), rescued the zebrafish knockdown phenotype, including movement defects, when expressed as either actin-dependent or -independent constructs (Hao et al., 2012; Lyon et al., 2014). As mentioned earlier, defects in RNA metabolism (Ling et al., 2013; Sleeman, 2013) and rescue by the actin-binding protein plastin 3 (Lyon et al., 2014) were observed in zebrafish models of *smn*. Combinations of genomics and morpholino knockdown demonstrated a link between *smn* and the adhesive molecule neurexin 2 (See et al., 2014) and with chondrolectin expressed in motor neurons (Sleigh et al., 2014).

### Chemical screens in zebrafish for SMA

In a recent study combining zebrafish, *Drosophila* and mouse models of SMA, dysregulation of ubiquitylation pathways caused β-catenin accumulation, and pharmacological inhibition of β-catenin by quercetin robustly ameliorated neuromuscular pathology (Wishart et al., 2014). Pharmacological or genetic suppression of ubiquitin-like modifier activating enzyme 1 (UBA1) was sufficient to recapitulate an SMA-like neuromuscular pathology in zebrafish, suggesting that UBA1 directly has an important role in disease pathogenesis. Tetrahydroindoles that alter the processing of amyloid precursor protein were tested in these various models and revealed that three of these compounds rescued motor neuron defects in *smn* morpholino knockdown zebrafish (Gassman et al., 2013). More generally, the results suggest that SMA could be an axonopathy (a disease in which the normal function of axons is disrupted), suggesting novel strategies for treating the disease. For example, pharmacological inhibition of pathways that are central to axonopathy have therapeutic potential as a treatment for SMA and many other neurological disorders in which axonal degeneration is a prominent feature.

## Amyotrophic lateral sclerosis (ALS)

### Background

ALS is a progressive neurodegenerative disorder characterised by the loss of both upper and lower motor neurons, leading to muscle weakness, paralysis and eventual death by respiratory failure. The age of onset is usually between 50 to 60 years of age and death typically occurs rapidly, 2–5 years after diagnosis (Kiernan et al., 2011). There is currently no cure for ALS and the only US Food and Drug Administration (FDA)-approved drug, riluzole, prolongs survival by only a few months (Miller et al., 2012). Around 90% of ALS cases are sporadic (sALS), with unknown aetiology (although retrospective studies have identified a small number of mutation-associated causes); the remaining 10% of cases are familial (fALS), with more than 20 genes implicated (Chen et al., 2013). Here, we focus on four causative genes, *SOD1*, *TARDBP*, *FUS* and *C9ORF72*, that account for more than half of fALS cases and for which zebrafish models have been developed (Table 1).

Superoxide dismutase 1 (*SOD1*) was the first gene linked to ALS (Rosen et al., 1993) and, to date, more than 170 mutations, affecting essentially every exon, have been reported (http://alsod.iop.kcl.ac.uk). Mutations in *SOD1* are responsible for ~20% of fALS and ~1–7% of sALS (Andersen and Al-Chalabi, 2011). Despite numerous mouse models of *SOD1*, 20 years later it is still unclear how mutations in *SOD1* lead to ALS, although a toxic gain-of-function mechanism has been identified (Boillée et al., 2006). SOD1 is a ubiquitously expressed, mainly cytoplasmic, anti-oxidizing enzyme and the toxicity of mutant SOD1 could be due to many causes (Ilieva et al., 2009).

The TAR-DNA binding protein (*TARDBP*) gene encodes the protein TDP-43, which was first linked to ALS as the major component of the cytoplasmic, ubiquitin-positive inclusions found in the majority of ALS cases, i.e. without mutations (Arai et al., 2006; Neumann et al., 2006). Mutations in *TARDBP* (Kabashi et al., 2008; Sreedharan et al., 2008) are responsible for ~3% of fALS and ~1.5% of sALS cases, and more than 40 mutations have been linked to ALS, the vast majority being located in the C-terminal glycine-rich region, which is responsible for protein-protein interactions that are as-yet unidentified (Lattante et al., 2013). TDP-43 is a mainly nuclear DNA- and RNA-binding protein and is involved in many steps of RNA processing, including transcription, splicing, RNA transport and stress-granule formation (Da Cruz and Cleveland, 2011), but ALS mutations in these functional regions are rare and their functional consequences are not understood.

Shortly after the discovery of *TARDBP* mutations in ALS, mutations in the gene fused in sarcoma (*FUS*; encoding the protein FUS) were identified in individuals with ALS (Kwiatkowski et al., 2009; Vance et al., 2009). More than 50 mutations of *FUS* have now been described in ALS and are responsible for ~5% of fALS and <1% of sALS (Lattante et al., 2013). These mutations are mostly clustered at the C-terminus, at a predicted nuclear-localisation signal (Ling et al., 2013). FUS is a mainly nuclear DNA- and RNA-binding protein. It shares a high functional similarity with TDP-43 and is also involved in several steps of RNA metabolism, including transcription and splicing (Lagier-Tourenne et al., 2012). The identification of *FUS* mutations soon after *TARDBP* highlighted the importance of altered RNA metabolism as a possible cause of ALS.

A huge (1000-fold) GGGGCC hexanucleotide repeat expansion in the non-coding first intron of chromosome 9 open reading frame 72 (*C9ORF72*) was identified in a large percentage of fALS cases (~40%), and was also observed in ~7% of cases of sALS (DeJesus-Hernandez et al., 2011; Renton et al., 2011). The function of the evolutionarily conserved protein is unknown, as is the mechanism of toxicity of the repeat expansions. However, recent evidence implicates the sequestration of RNA-binding proteins in RNA foci formed by sense and antisense repeat-containing *C9ORF72* RNA, as well as repeat-associated non-ATG translation of both sense and antisense transcripts, leading to the expression of six expansion proteins that form cytoplasmic and intranuclear inclusions, although the toxic effect of haploinsufficiency is still debated (Gendron et al., 2014).

### Zebrafish models of ALS

Although zebrafish models of *SOD1* toxic gain-of-function mutations appeared much later than mouse and rat models, they have nonetheless contributed recently to our understanding of the cellular and neuronal dysfunction of the disease. In 2007, Lemmens and colleagues demonstrated that overexpression of human mutant *SOD1^A4V^*, *SOD1^G93A^* or *SOD1^G37R^* mRNA resulted in aberrant ventral root projections in zebrafish embryos (Lemmens et al., 2007), resembling mouse models of *SOD1* in which an early retraction of presynaptic motor endings, known as ‘dieback’, is observed prior to the death of motor neurons (for review, see Dadon-Nachum et al., 2011; Murray et al., 2010), indicating that early, notable changes occur at the NMJ long before clinical presentation of ALS, which could also be the case for individuals with ALS (Dadon-Nachum et al., 2011). This was the first dominant gain-of-function neurodegenerative disease model to be studied in zebrafish, and validated the use of this animal model for experimental investigation while providing evidence that early changes in neuronal structure could arise *in vivo* following expression of mutant proteins associated with SOD1 pathology. Stable transgenic mutant lines of zebrafish *sod1* (G93R) (Ramesh et al., 2010; Sakowski et al., 2012) have been generated more recently, including useful *sod1* (G93R and G85R) heat-shock reporter lines (McGown et al., 2012). These models display hallmarks of ALS that include an age-related decrease in swim endurance, atrophy of locomotor muscles, paralysis with concomitant loss of NMJ integrity and, in some cases, early death.

Sharp electrode recordings from the motor neuron cell body of postnatal (6- to 10-days old) presymptomatic *SOD1^G85R^* mice have revealed hyperexcitability in motor neuron populations in the spinal cord (Bories et al., 2007). An advantage of the larval zebrafish is that it lends easily to electrophysiological investigations not only at the periphery (NMJ) but also centrally, within the spinal cord. A recent report has demonstrated that, in a stable transgenic *SOD1^G93R^* zebrafish, spontaneous miniature glycinergic excitatory postsynaptic currents (mEPSCs) onto motor neurons occur at a lower frequency than in motor neurons of wild type and zebrafish expressing human wild-type *SOD1* (McGown et al., 2012). Although the cause of this reduction has not been elucidated, the authors propose that the early reductions in the strength of this synapse onto motor neurons, particularly at early stages in development when synaptic connections are forming, might have lasting consequences whereby even slight increases in excitability during adulthood can promote motor neuron stress (McGown et al., 2012).

In addition to *SOD1*, models for the more recently (since 2008) identified ALS genes *TARDBP*, *FUS* and *C9ORF72* have also been developed in the zebrafish. For these genes, the role of loss of function, toxic gain of function or perhaps their combination is still under evaluation in the field. Consequently, models for both genotypes have been developed, although mostly by transient approaches because knockouts and stable transgenic lines have taken longer to develop. Mutations in the *TARDBP* gene were identified in individuals with both familial and sporadic forms of ALS (Kabashi et al., 2008) and, soon after, a transient zebrafish genetic model was developed (Kabashi et al., 2010b; Sreedharan et al., 2008). In this model, expression of human *TARDBP* mRNA containing one of three missense mutations (*TARDBP*^G348C^, *TARDBP*^A315T^ or *TARDBP*^A382T^), but importantly not wild-type *TARDBP* at the same level of expression (which is a problem in other models) (see Wegorzewska and Baloh, 2011), resulted in locomotor defects and hyperbranched ventral root projections to trunk musculature, as seen with zebrafish *SOD1* models. Furthermore, it was demonstrated that the knockdown of the zebrafish *tardbp* gene also resulted in locomotor and ventral root defects, which could be rescued by co-injection with wild-type *TARDBP* mRNA but not mutant mRNA (Kabashi et al., 2010b). Given the limitations of the transient approaches used to generate these models, however, the results from these albeit independent studies need to be interpreted with caution. Interestingly, increased NMJ denervation (possibly due to dieback) has also been reported in rat and mouse models of TDP-43 mutations (Swarup et al., 2011; Zhou et al., 2010). In a follow-up study, expression of *TARDBP*^G348C^ mRNA in zebrafish resulted in impaired neuromuscular synaptic transmission, reduced frequency of miniature endplate currents (mEPCs), reduced quantal transmission, orphaned presynaptic and postsynaptic structures at the NMJ, and motor neuron death following exposure to the glutamatergic agonist N-methyl-D-aspartate (NMDA) (Armstrong and Drapeau, 2013a).

These initial studies of *TARDBP* utilized transient genetic models of zebrafish orthologues. Recent efforts to generate *tardbp* knockout lines in zebrafish have highlighted an unexpected subtlety associated with the use of this model organism. Unlike mammals, zebrafish possess a paralogue of *tardbp* [Tar DNA binding protein-like (*tardbpl*)], which lacks the C-terminal glycine-rich region, presumably owing to a chromosome duplication event deep in its ancestral evolution (Postlethwait et al., 2000). Interestingly, a novel splice variant of *tardbpl* has been shown to recover loss of *tardbp* following gene disruption of the latter with ZFNs (Schmid et al., 2013), as well as in another fish line where a premature stop codon (Y220X) in *tardbp* was isolated by TILLING (Hewamadduma et al., 2013). In both studies, inactivation of both *tardbp* and *tardbpl* (the latter either by gene disruption by ZFNs or by knockdown) resulted in a severe phenotype and early lethality, although the phenotype of double heterozygotes has not been reported and might more closely resemble the ALS genotype (if due to haploinsufficiency) and perhaps the (partial) knockdown phenotype. This strong *tardbp/tardbpl* phenotype is highly reminiscent of *Drosophila* knockout models of the related *tbph* gene, which display early lethality in the second instar stage, and *Tardbp* knockout mice, which fail to implant during embryogenesis (Sephton et al., 2010; Voigt et al., 2010; Wu et al., 2010), confirming the important role that TDP-43 plays during developmental processes in disparately related organisms. However, caution is necessary when extrapolating these findings to ALS, because individuals with (heterozygous) mutations in *TARDBP* possess a wild-type copy of *TARDBP* in addition to the allele containing a missense mutation, both of which are expressed throughout the progression of this disease. In addition, it might not be the loss of function owing to haploinsufficiency but rather a toxic gain of function owing to missense mutations that causes the disease. Thus, it would be ideal to generate a heterozygous zebrafish model, with both a mutant and a wild-type allele, but a conditional gain of toxic function might be necessary if this proves lethal.

Although less studied, zebrafish models of other genes associated with ALS have been developed. Injection of human mutant *FUS* mRNA (P525L) resulted in disrupted nuclear import (Dormann et al., 2010) and accumulation in cytosolic stress granules (Bosco et al., 2010), and either mRNA or morpholino injection resulted in locomotor deficits and ventral root projection abnormalities (Kabashi et al., 2011a). Recent work has demonstrated that transient mutant human *FUS^R521H^* expression resulted in pathological hallmarks of ALS at the zebrafish NMJ, which include reduced fidelity of synaptic transmission, reduced quantal content and abnormal synaptic morphology (Armstrong and Drapeau, 2013b), as for *TARDBP*. FUS and TDP-43 share some structural similarities, and evidence that they might act in a molecular pathway that is separate from SOD1, with TDP upstream from FUS, has been demonstrated by epistasis analyses in zebrafish (Kabashi et al., 2011a) and in *Drosophila* (Wang et al., 2011). However, lines with *fus* knockout or stable *FUS* transgenes are needed to advance studies using these models.

The recently reported hexanucleotide repeat expansion in *C9ORF72* that has been attributed to a large proportion of fALS and sALS cases (DeJesus-Hernandez et al., 2011; Renton et al., 2011) has garnered substantial interest among both ALS and frontotemporal lobar degeneration researchers. There are no published mammalian models of *C9ORF72*, but a recent knockdown model of the zebrafish orthologue, *c13h9orf72*, demonstrated impaired locomotor activity and abnormal ventral root projections (Ciura et al., 2013). Because there is considerable interest in this gene of unknown function, undoubtedly much attention will be focused on elucidating the molecular and functional role it plays in conferring disease, in particular the possible toxic effects of the expansion in transgenic lines.

### Chemical screens in zebrafish for ALS

Studies of a *sod1* mutant in zebrafish opened the possibility of screening small molecules for recovery from the phenotype. A neuronal-stress reporter line (McGown et al., 2013) was used to validate the effectiveness of riluzole in this model, the only drug currently used to treat ALS clinically, and olesoxime and apomorphine-S were found to be effective in an oxidative stress assay (Da Costa et al., 2014). One possible explanation for the anomalies described above occurring at the NMJ in zebrafish expressing mutant TDP-43 is reduced calcium ion entry in the presynaptic terminal during the action potential; these defects were completely prevented in larvae expressing mutant human *TARDBP* treated with L-type calcium channel agonists (Armstrong and Drapeau, 2013a).

**Fig. 2.**
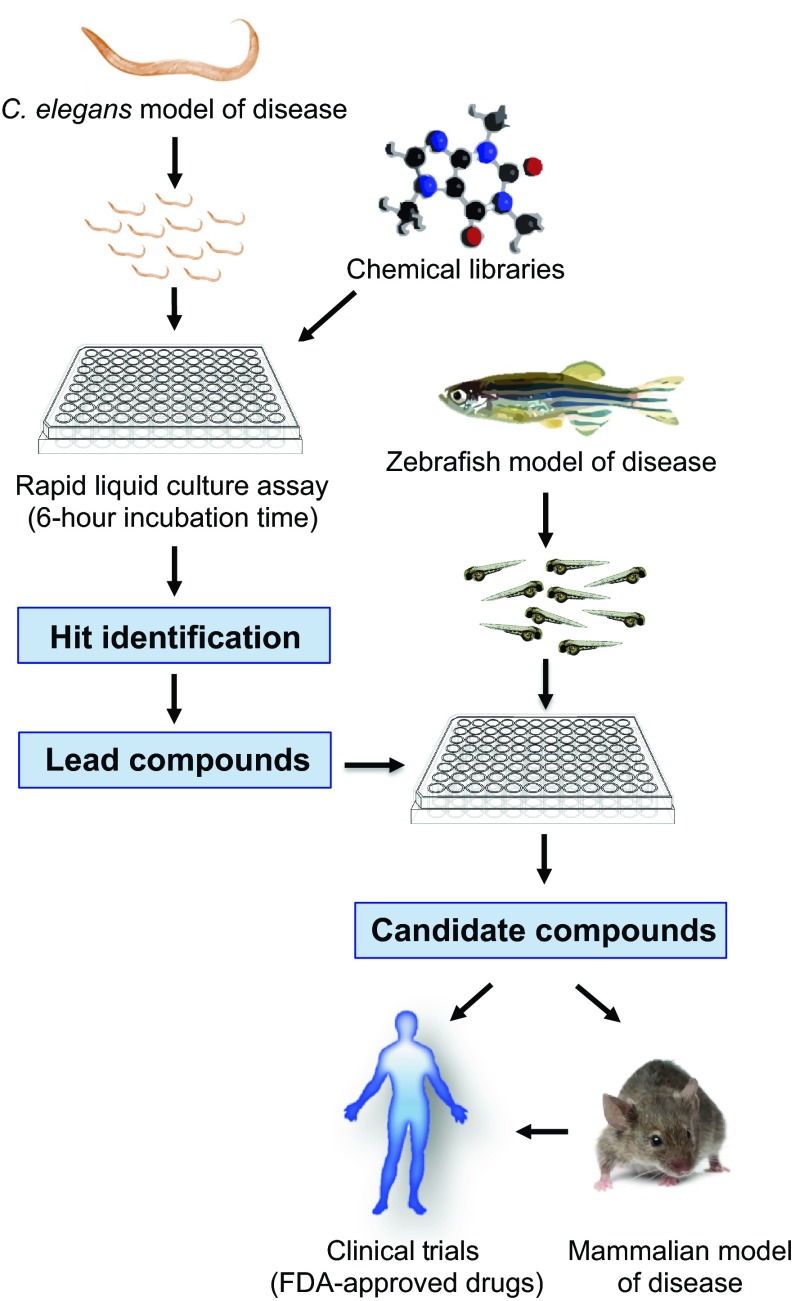
**Motor neuron disorders: from bench to clinic.** One proposed strategy for drug discovery for MNDs (and other diseases) is to first start a large and rapid chemical screen in *C. elegans* and confirm the positive hits in a zebrafish. Once positive compounds that reverse or ameliorate the disease phenotype in both models are encountered, they can then be tested in mammal systems and/or go straight to clinical trials, if the compounds are already approved for safety and efficacy by the FDA.

As with studies of SMA combining zebrafish with other models, our strategy for drug discovery in ALS was to start with a chemical screen in a *Caenorhabditis elegans* model and then confirm the positive hits in a zebrafish model. Once compounds that reverse or ameliorate the disease phenotype are identified, they can be tested in mammalian systems and/or in clinical trials for FDA-approved compounds (Fig. 2). Worms grown in liquid culture exhibit a thrashing behaviour that is more vigorous than crawling on plates and we have found that this greatly accelerates neuronal dysfunction in transgenic models of ALS mutations (Vaccaro et al., 2012b). As a result, paralysis is manifested in a matter of hours in liquid culture instead of days as on solid agar plates, thus permitting a rapid, large-scale chemical screening assay in *C. elegans*. Starting with an *in vivo* pilot chemical screen using this strategy, we successfully demonstrated that our *C. elegans* and zebrafish ALS mutation models could be used to identify neuroprotective molecules. This, the first *in vivo* chemical genetic screening platform for ALS genes (Vaccaro et al., 2012a), revealed methylene blue (MB) as a potent suppressor of mutant TDP-43 and mutant FUS motor neuron toxicity *in vivo*. In both worms and fish, MB prevented motor deficits and reduced the level of oxidative stress observed upon the expression of mutant proteins. In a subsequent study, we showed that compounds structurally related to MB, such as salubrinal, guanabenz and the novel compound phenazine, are also potent suppressors of mutant TDP-43 proteotoxicity (Vaccaro et al., 2013). Our experiments demonstrated further that all these neuroprotective compounds function within the unfolded protein response during ER stress and they operate through different branches of this pathway to achieve neuroprotection (Vaccaro et al., 2013).

We have capitalized on the strength of our *in vivo* assays to screen large chemical libraries in the search for potential therapeutic drugs for ALS. We recently isolated a number of chemical suppressors of mutant TDP-43 toxicity (our unpublished data) and in particular we observed that several neuroleptics protect against development of the motor phenotype. The FDA-approved drug that emerged from this screen offers an exciting starting point for further ALS research because one can work back to identify the target(s) of positive hits and investigate their mode of action to better understand the mechanism of disease pathogenesis. In addition to ‘repurposing’ drugs identified as hits, it is possible to screen novel structural analogues to develop more selective therapeutics. Thus, eventually, it should be possible to identify better drug targets and develop more potent disease modifiers.

## Hereditary spastic paraplegia (HSP)

### Background

HSP designates a set of inherited heterogeneous disorders characterised by lower extremity weakness and spasticity caused by the progressive degeneration of the upper motor neurons. There is currently no cure, but several drugs are available to alleviate some of the symptoms, e.g. spasticity (Fink, 2013). More than 50 different loci have been discovered, designated SPG (spastic paraplegia) and numbered by order of discovery, with some 60 genes identified to date. Many (~40%) forms of autosomal dominant HSP are due to mutations in *SPG4*, encoding spastin, a ubiquitously expressed, mainly cytosolic protein that has microtubule-severing properties (Blackstone, 2012). Mutations in *SPG3A* (atlastin-1) account for ~10% of autosomal dominant HSP and are the second most common cause of HSP as well as the most common form of early-onset HSP; this form is usually clinically described as uncomplicated (Namekawa et al., 2006). Atlastin is a GTPase important for the formation of tubular ER, where it also interacts with spastin (Blackstone, 2012). *SPG11* (spatacsin) mutations occur for ~21% of autosomal recessive cases of HSP, and all except one of the 100 mutations identified in this gene to date lead to truncation of the protein, indicating a possible loss of function (Stevanin et al., 2008). Mutations in *SPG8* (strumpellin; also known as KIAA0196) have also been reported. They account for ~4% of HSP and are inherited in an autosomal dominant manner (Valdmanis et al., 2007). Strumpellin has been identified as a subunit of the Wiscott-Aldrich syndrome protein and SCAR homolog complex (WASH complex) (Blackstone, 2012). Other less common HSP-associated genes are *Alsin*, *SPG42* (*SLC33A1*), *SPG39* (*PNPLA6*), *SPG53* (*VPS37A*), *SPG46* (*GBA2*) and *SPG43* (*C19ORF12*) (Fink, 2013; Landouré et al., 2013; Martin et al., 2013; Yoon et al., 2013). A recent exome sequencing study identified 18 new putative HSP genes (Novarino et al., 2014). The HSP proteins are involved in many different cellular functions, which underlie the pathogenic mechanisms by which mutations will cause HSP, including disrupted axonal pathfinding and axonal transport, disrupted ER morphology, myelin abnormality, mitochondrial dysfunction, disturbed endosomal dynamics and disturbed lipid metabolism (Blackstone, 2012).

### Zebrafish models of HSP

Zebrafish have been widely used to validate the pathogenic nature of genes involved in HSP, with over 40 publications to date (more than for SMA or ALS), although all are studies of transient knockdown because no genetic models of HSP have yet been generated. Most HSP genes cause disease in an autosomal recessive fashion, thus implying loss-of-function and dominant-negative molecular mechanisms, which can be readily modelled at a genetic level in zebrafish. However, because zebrafish lack upper motor neurons, the phenotypes reflect at best a general effect rather than one of a specific nature. A number of reports have convincingly shown that the loss of function of HSP-associated genes lead to major motor neuron defects, usually with impaired locomotion, consistent with the roles of spastin and other less common HSP genes that are involved in axonal transport (Blackstone, 2012). For instance, knockdown of spastin (*SPG4*) caused dramatic defects in spinal motor axon outgrowth, abnormal motility and perturbation of axonal microtubule dynamics (Wood et al., 2006; Butler et al., 2010; Wood et al., 2006). Loss of function of atlastin (*SPG3A*) severely impaired spinal motor axon architecture and fish motility (Fassier et al., 2010). However, because autosomal dominant mutations of *spastin* and *atlastin* are (major) causes of HSP, rather than loss of function, caution is necessary when interpreting these knockdown models in zebrafish. Motor axons in fish injected with *strumpellin* (*SPG8*) morpholino were also shorter and had abnormal branching and impaired motility, which were rescued upon co-injection of wild-type human *SPG8* mRNA but not mRNA with HSP-related variants, validating the latter as loss-of-function mutations (Valdmanis et al., 2007). Knockdown of either *spatacsin* (*SPG11*) or *spastizin* (*SPG15*) severely compromised branching of spinal cord motor neurons at the NMJ and caused locomotor impairment (Martin et al., 2012). Knockdown of *slc33A1* [encoding Solute carrier family 33 (acetyl-CoA transporter), member 1; *SPG42*) in zebrafish embryos also caused a tail curvature phenotype and abnormal axon outgrowth from the spinal cord (Lin et al., 2008). Knockdown of *gba2* (Glucosidase beta 2; *SPG46*) led to abnormal locomotion and outgrowth of spinal motor axons (Martin et al., 2013). *pnpla6* (*SPG39*) knockdown resulted in developmental abnormalities and motor neuron defects, including axon truncation and branching (Song et al., 2013). A loss of motility was induced in *vps37a* (Vacuolar protein sorting 37A; *SPG53*) knockdown zebrafish larvae (Zivony-Elboum et al., 2012). In a number of cases, these deficits could be rescued using the human orthologous mRNA transcript, but not using the disease-causing mRNA transcript, arguing for loss, rather than toxic gain, of function.

*Alsin* (*ALS2*) is a gene that is frequently mutated in juvenile HSP and ALS through an autosomal recessive mode of action, which leads, in most cases, to protein truncation. Similarly to *spastin* and *strumpellin* models, knockdown of *alsin* causes shortening of motor axons and a loss of neurons in the spinal cord (Gros-Louis et al., 2008). It is important to note that a number of groups generated *Als2* knockout mice in which the first or the second exon of the mouse *alsin* orthologue is deleted, but none of them observed deficits associated with motor neuron degeneration (Deng et al., 2007; Yamanaka et al., 2006). The motor phenotype in zebrafish caused by knockdown of *als2* by targeting the initial ATG was partially rescued upon overexpression of an alternative transcript whose initiating ATG is located within the fourth exon of *ALS2*. This result suggests that the lack of deficit in the knockout murine models, which relied on the deletion of only the initial exons of this gene, could be attributable to an analogous initiation of translation further downstream (Gros-Louis et al., 2008). This example of incomplete knockout in mice versus comprehensive knockdown in zebrafish illustrates the utility of zebrafish for obtaining a more complete disruption of gene function in some cases.

Finally, a recent exome sequencing study identified 18 new putative HSP genes, four of which (*arl6Ip1*, *mars*, *pgap1* and *usp8*) had morpholino knockdown phenotypes in zebrafish (Novarino et al., 2014). Whereas knockdown of *mars* generated a severe phenotype, the others, including *arl6Ip1* (associated with the ER) and *usp8* (involved in endosomal sorting), yielded knockdown defects in touch-induced and spontaneous motor behaviour as well as motor axon morphology that are relevant to HSP, establishing the power of this combination of human genomics and zebrafish knockdowns for the identification and validation of disease-related variants, respectively. It is worth noting, however, that genetic knockout has not yet been reported for any HSP gene and will be necessary to help advance studies of this disorder using zebrafish models.

So far, no drug test or screens for HSP using zebrafish have been published. Nonetheless, the validation of *strumpellin* mutations (Valdmanis et al., 2007) has allowed the development of a patented diagnostic test for HSP that is licensed to Athena Diagnostics Inc., emphasising the usefulness of variant validation in zebrafish. With the urgent need to develop effective treatments for these diseases, we encourage further drug discovery campaigns for all MNDs using a similar approach to that proposed in Fig. 2 to rapidly identify lead compounds.

## Chemical screens in zebrafish models for other neural diseases

Successful efforts in developing therapeutics with other zebrafish neurological disease models bode well as examples for approaches that could be developed for MNDs. In a recent paper, a phenotype-based screen identified clemizole (an FDA-approved compound) as a potential lead compound for Dravet syndrome because it effectively inhibited spontaneous convulsive behaviours and electrographic seizures in zebrafish *scn1Lab* mutants (Baraban et al., 2013). In another successful *in vivo* screen study, using the zebrafish lateral line sensory system as a model system for mammalian auditory hair cells, the collaborative work of the Rubel and Raibel labs identified two small molecules (named PROTO-1 and PROTO-2) (Owens et al., 2008) and an FDA-approved compound (named tacrine) (Ou et al., 2009) that protect hair cells from aminoglycoside-induced death. These compounds also inhibited hair cell death in the mammalian inner ear and subsequently preserved hearing. In a screen of the commercially available Prestwick chemical library, three small molecules were identified that ameliorated muscle pathology and increased survival in a zebrafish model of Duchenne muscular dystrophy (*dystrophin*-null) (Kawahara et al., 2011). Interestingly, one of the chemicals, aminophylline, a nonselective phosphodiesterase (PDE) inhibitor, demonstrated a promising capacity to restore normal muscle structure and upregulate the cAMP-dependent protein kinase A (PKA) pathway in dystrophin-deficient fish (Kawahara et al., 2011). Because similar approaches are underway for SMA and ALS zebrafish models, and could be implemented for HSP, these successful screens in neurological models open the exciting possibility of drug discovery for MNDs, where therapeutic interventions remain severely limited and are in great need of development.

## Concluding remarks and perspectives

To date, over a hundred papers have shown that various pathological aspects of MNDs caused by expression of mutated genes can be modelled in the zebrafish. This model is now poised at the beginning of a new age of genome manipulation and the new editing approaches will undoubtedly improve the ability to generate more and better MND models in zebrafish. These models can then be used to study the molecular basis of disease and they will certainly provide novel and useful insights into MNDs. The ability to simultaneously target multiple genes and in different combinations in a single embryo, such as using combinations of morpholinos and mRNAs (Kabashi et al., 2011b; Kabashi et al., 2010a) and by improved genome-editing approaches as reported for the CRISPR/Cas system (Jao et al., 2013) and the *Gal4/UAS* approach (Liu and Leach, 2011a), represents an attractive strategy for the study of epistasis in MNDs, an important aspect for the analysis of complex diseases that is not easily implemented in other models such as mice. Of general interest for MND models, a recent knockdown screen for modifiers of ALS-related genes in zebrafish identified *Epha4* as a gene that, when knocked down, could rescue the phenotype caused by expression of mutant *SOD1* or *TARDBP*. Upon co-knockdown of *SMN1*, *Epha4* also increased survival of mouse and rat models of ALS (Van Hoecke et al., 2012). Furthermore, *EPHA4* expression in individuals with ALS inversely correlated with disease onset and survival, and loss-of-function mutations were associated with long survival. These results, starting with zebrafish, then validated in mammalian models and ultimately correlated with patient aetiology, nicely indicate the power of the zebrafish model for providing translational insights into MND.

The opportunity to apply discovery-driven approaches *in vivo* in a vertebrate and design high-throughput screens, enabling novel hypotheses to be subsequently tested in mammalian models or in clinical studies, is a major strength of the zebrafish model. Although, to date, there is not a single ‘positive hit’ published from a zebrafish-based drug screen for neurological disorders that has entered a Phase 1 clinical trial, the protective compounds discovered in the recent studies described above are very promising leads for future investigation. The prospect of using zebrafish for drug discovery opens new avenues for discovering treatments for MNDs.
